# Editorial: Novel mechanisms and approaches in kidney/pancreas-kidney transplant-related injury

**DOI:** 10.3389/fimmu.2022.1082590

**Published:** 2022-11-22

**Authors:** Nianqiao Gong, Yuanyuan Zhao, Hua Zhu, Chenhui Wang

**Affiliations:** ^1^ Institute of Organ Transplantation, Tongji Hospital, Tongji Medical College, Huazhong University of Science and Technology, Key Laboratory of Organ Transplantation of Ministry of Education, National Health Commission and Chinese Academy of Medical Sciences, Wuhan, China; ^2^ Division of Cardiac Surgery, Department of Surgery, The Ohio State University Wexner Medical Center, Columbus, OH, United States; ^3^ The Key Laboratory for Human Disease Gene Study of Sichuan Province and the Department of Laboratory Medicine, Sichuan Provincial People’s Hospital, University of Electronic Science and Technology of China, Chengdu, China

**Keywords:** transplantation, allograft injury, epigenetic regulation, posttransplant complications, mechanism, approach

Protecting graft function, modulating immune rejection, and ameliorating complication-induced damage are essential for successful transplantation.

Ischemia–reperfusion injury (IRI) represents a main obstacle for organ protection during the maintenance of donor organ, procurement, and grafting and can directly impair organ function. IRI can ultimately result in delayed graft function (DGF) and even primary non-function (PNF), thus increasing the risk of rejection and reducing the survival of grafts and recipients (1). Unfortunately, although many previous studies have focused on the mechanisms and treatments of IRI, there is still no definitive guidance for the prevention and treatment of IRI, thus creating the need for dedicated research (2). Previous research, which focused mostly on signal transduction and organelle homeostasis, led to the administration of current medicines, novel chemical compounds, and natural products (3–6). In the current Research Topic, Ren et al. demonstrated that estradiol, an evolutional intrinsic sex hormone, can exert renoprotective functionality against IRI *via* its receptor and the transforming growth factor type I receptor-SMAD (TGF-βRI-SMAD) pathway. Another interesting development relates to epigenetic regulation in kidney transplantation. In their review, Xiang et al. report that DNA methylation plays roles in the progression of acute tubular necrosis during IRI and DGF and can play roles in the development of renal graft interstitial fibrosis/tubular atrophy (IF/TA) and the inhibition of kidney repair. Furthermore, histone modification and noncoding RNAs (ncRNAs) are related to damage in the proximal tubules *via* a range of mechanisms, including mitochondria and exosomes. Remarkably, epigenetic regulation is also closely associated with diabetic kidney disease. The diagnosis, effects, and treatment of epigenetic regulation in kidney transplantation provide researchers with significant opportunities for future research.

T cell-mediated rejection (TCMR) and antibody-mediated rejection (ABMR) constitute alloimmune rejection following transplantation. Thus, gaining a better understanding of the regulatory mechanisms underlying TCMR and ABMR has been a significant goal in the organ transplantation field for decades (Juneja et al.). The production of adenosine from extracellular ATP mediated by CD39 and CD73 may represent a mechanism that could be used to shape regulatory T cells (Tregs). Tripathi et al. report the upregulation of both CD39 and CD73 in expanded antigen-specific Treg-enriched lines (ASTRLs) that also exhibited the extracellular hydrolysis of ATP. These authors demonstrated that the adenosinergic pathway plays a major role in the regulatory effects in kidney transplant patients, thus suggesting that combining the infusion of Tregs with adenosine receptor agonists or increasing the expression of CD39 expression in grafts may enhance the regulatory response to the allograft and achieve tolerance.

ABMR has drawn significant attention, particularly in highly sensitized kidney donor recipients. Allograft biopsies with lesions of microvascular inflammation (MVI) along with the simultaneous evaluation for anti-human leukocyte antigen (HLA) donor-specific antibodies (DSAs) are now widely recognized based on the classification developed by the Banff 2019 Working Group (7). Once diagnosed, the recipient would be given systemic treatments to reduce DSAs and their sources (B cells and plasma cells). Considering its severity with regard to clinical outcome, the prediction of ABMR has become an urgent necessity. Wu et al. investigated whether the B cell-activating factor (BAFF) could represent a noninvasive biomarker of ABMR in kidney transplantation but found that serum soluble BAFF is not an appropriate diagnostic biomarker for ABMR, although the levels of this factor at 3 months posttransplantation did have a predictive value for DSA/ABMR. In addition to anti-HLA antibody, anti-blood type antibody is a key issue for ABO-incompatible (ABOi) kidney transplantation (8). In the case report by Hayashi et al., five ABO blood type O recipients received kidney grafts from wives with blood type B. Flow-cytometry T cell crossmatch (FTXM) was positive, but complement-dependent cytotoxicity crossmatch was negative. In one case, an eluate prepared from the donor’s T lymphocytes agglutinated only type B red blood cells, thus implying the existence of blood type B substances on the donor T lymphocytes. After removing type B antibodies, FTXM became negative for all five patients. This interesting report enhances our understanding of how transplant-related antibodies work.

Xiang et al. reviewed the processes by which epigenetic regulation can modulate immune cells during transplantation. The demethylation of Foxp3 was found to be associated with intragraft higher expression levels of Tregs, while the hypermethylation of *PDCD1* in CD27^-^ memory CD8^+^ T cells showed a positive correlation with acute rejection. In addition, hypermethylated genes were found to be enriched in the T cells of peripheral blood mononuclear cells (PBMCs) and mechanistic target of rapamycin signaling (mTOR) pathways in acute rejection (AR)-related graft dysfunction. Furthermore, the DNA methylation profiles of operational tolerant patients showed demethylation of CD20-encoding genes, thus resulting in the survival of transitional B cells. Non-histone acetylation of *Foxp3*, *SIRT1*, and Zn-dependent *HDACs* led to the stabilization of the phenotype and functionality of Tregs. ncRNAs [microRNA (miRNA), long noncoding RNA (lncRNA)], regardless of whether they are derived from immune cells or not, may regulate their target genes and therefore modulate T helper cells, dendritic cells, Th1/Th2 cells, and ABMR. Research efforts aiming to identify ncRNAs as potential biomarkers for transplantation have advanced significantly over recent years.

When considering posttransplant complications, pathogenic infections represent the most common threat to the survival of grafts and recipients. In a manner that differs from cytomegalovirus (CMV), *Pneumocystis carinii* (PC), and human parvovirus B19, which damage extrarenal organs more often than the kidney, infection with BK polyomavirus (BKV) can readily cause insult to the kidney and is becoming increasingly more common. BKV-associated nephropathy (BKVN) may destroy kidney function in only a short time period (9).

Using dynamic Cox regression, Fang et al. identified several factors that were positively correlated with BKV reactivation, including advanced age, the combination of basiliximab with cyclophosphamide, acute graft rejection, a higher body mass index, estimate glomerular filtration rate (eGFR), urinary protein level, urinary leukocyte level, and blood neutrophil count. In addition, male gender and a higher serum albumin level and platelet count served as protective factors during the first year after transplantation. Meanwhile, Wen et al. evaluated the efficacy of programmed monitoring for graft-derived cell-free DNA (GcfDNA) to identify BKVN in 158 kidney transplant recipients. These authors verified the diagnostic performance of GcfDNA alterations between programmed monitoring and the time of biopsy-proven BKVN. This research focused on how to predict and diagnose BK infection, although other issues also need to be elucidated. For example, it is important for us to identify methods to balance rejection and infection during immunosuppressant reduction treatment (10). We also need to ascertain the correlations between the titer of BK virus in the serum/urine and transplant outcome and develop methods to treat BKV infection.

In general, protecting graft function, modulating immune rejection, and ameliorating the damage caused by complications are all processes that can interact with each other (Tammaro et al.; Quaglia et al.; 11). Donor injury can induce innate and adaptive immune reactions, while rejection can exacerbate graft damage. Furthermore, complications can impair grafts and trigger rejection, while the worsening status of grafts and recipients can aggravate complications. To achieve preferable outcomes in kidney/pancreas-kidney transplantation, novel mechanisms and approaches need more basic and clinical exploration.

## Author contributions

NG, YZ, HZ and CW wrote and revised the manuscript, have contributed equally to this work, and approved it for publication. All authors contributed to the article and approved the submitted version.

## Conflict of interest

The authors declare that the research was conducted in the absence of any commercial or financial relationships that could be construed as a potential conflict of interest.

## Publisher’s note

All claims expressed in this article are solely those of the authors and do not necessarily represent those of their affiliated organizations, or those of the publisher, the editors and the reviewers. Any product that may be evaluated in this article, or claim that may be made by its manufacturer, is not guaranteed or endorsed by the publisher.
